# Genetic Variation in Drought-Tolerance Traits and Their Relationships to Growth in *Pinus radiata* D. Don Under Water Stress

**DOI:** 10.3389/fpls.2021.766803

**Published:** 2022-01-04

**Authors:** Ahmed Ismael, Jianming Xue, Dean Francis Meason, Jaroslav Klápště, Marta Gallart, Yongjun Li, Pierre Bellè, Mireia Gomez-Gallego, Ki-Taurangi Bradford, Emily Telfer, Heidi Dungey

**Affiliations:** ^1^Scion (New Zealand Forest Research Institute Ltd.), Rotorua, New Zealand; ^2^Research and Development, Livestock Improvement Corporation, Hamilton, New Zealand; ^3^Scion (New Zealand Forest Research Institute Ltd.), Christchurch, New Zealand; ^4^Centre for Planetary Health and Food Security, Griffith University, Nathan, QLD, Australia; ^5^Agriculture Victoria, AgriBio Center, Bundoora, VIC, Australia; ^6^INRAE, IAM, Université de Lorraine, Nancy, France

**Keywords:** drought tolerance, water stress, heritability, genetic correlation, genomic selection, carbon isotope composition, *Pinus radiata*, chlorophyll fluorescence

## Abstract

The selection of drought-tolerant genotypes is globally recognized as an effective strategy to maintain the growth and survival of commercial tree species exposed to future drought periods. New genomic selection tools that reduce the time of progeny trials are required to substitute traditional tree breeding programs. We investigated the genetic variation of water stress tolerance in New Zealand-grown *Pinus radiata* D. Don using 622 commercially-used genotypes from 63 families. We used quantitative pedigree-based (Genomic Best Linear Unbiased Prediction or ABLUP) and genomic-based (Genomic Best Linear Unbiased Prediction or GBLUP) approaches to examine the heritability estimates associated with water stress tolerance in *P. radiata*. Tree seedling growth traits, foliar carbon isotope composition (δ^13^C), and dark-adapted chlorophyll fluorescence (Y) were monitored before, during and after 10 months of water stress. Height growth showed a constant and moderate heritability level, while the heritability estimate for diameter growth and δ^13^C decreased with water stress. In contrast, chlorophyll fluorescence exhibited low heritability after 5 and 10 months of water stress. The GBLUP approach provided less breeding value accuracy than ABLUP, however, the relative selection efficiency of GBLUP was greater compared with ABLUP selection techniques. Although there was no significant relationship directly between δ^13^C and Y, the genetic correlations were significant and stronger for GBLUP. The positive genetic correlations between δ^13^C and tree biomass traits under water stress indicated that intraspecific variation in δ^13^C was likely driven by differences in the genotype’s photosynthetic capacity. The results show that foliar δ^13^C can predict *P. radiata* genotype tolerance to water stress using ABLUP and GBLUP approaches and that such approaches can provide a faster screening and selection of drought-tolerant genotypes for forestry breeding programs.

## Introduction

Global climate change scenarios predict that large areas of planted forests will be at risk in the future due to warmer temperatures and changing precipitation patterns and drought (IPCC, 2013). Drought can cause severe negative effects on plant growth and survival (Adams et al., 2009; Allen et al., 2010), such as increased susceptibility to pathogens by reducing the amount of resources available for defense (Kolb et al., 2016) and alter the forest stand dynamics by shifting species structure and composition (Rigling et al., 2013). Increases in the intensity, duration and frequency of drought are often associated with a significant increase in tree mortality in forest ecosystems across the globe (Allen et al., 2010). In New Zealand, future scenarios predicts an increase of drought frequencies in the northern and eastern regions of the country (Mullan et al., 2005).

The New Zealand commercial forestry sector is the third-largest primary sector export earner (New Zealand Forest Owners Association [NZFOA], 2019). Planted forests of *Pinus radiata* D. Don comprise 90% of the commercial forest land and cover 6.4% (1.7 million ha) of New Zealand’s land area (Forest Owners Association, 2016). The increased frequency and severity of drought periods are a potential major risk to the productivity and health of New Zealand forests (Dunningham et al., 2012). Previous modeling studies have estimated that drought-induced mortality in New Zealand forests could decrease the productivity of *P. radiata* plantations on average by16 m^3^ ha^–1^ Watt et al. (2010) and cause an equivalent of $38 M yr^–1^ loss in Eastern New Zealand forests alone (Xue et al., 2012). Modeling of future climates showed that a 2°C rise in global temperatures could reduce *P. radiata* productivity in New Zealand by at least 10% by 2080, due to low precipitation and pathogen outbreaks (Meason and Mason, 2014). One approach to mitigate the impact of climate change and drought periods on *P. radiata* is the selection and deployment of genotypes less susceptible to water-limiting conditions. Drought-tolerant genotypes require less water for wood biomass production and tolerate drier environments compared to more sensitive genotypes. Previous comparative studies of water-use efficiency across *P. radiata* genotypes have found contradictory results; and differences in water use between genotypes range from none (Whitehead et al., 1983; Hatton and Vertessy, 1990; Walcroft et al., 1996; Li et al., 2015; Espinoza et al., 2017) to large (Bennett and Rook, 1978; Rodríguez-Gamir et al., 2019) across different experimental conditions. The selection and breeding of drought-tolerant forest tree species is being investigated worldwide (e.g., Pita et al., 2005; Roussel et al., 2009; Gaspar et al., 2013; Marguerit et al., 2014; Chakhchar et al., 2017), although the genetic basis of drought tolerance is not well understood.

Drought can be mitigated by increasing water-use efficiency (WUE) at the whole plant level. This is defined as the ratio between biomass production and water consumption. At the leaf level, intrinsic water-use efficiency (iWUE) is described as the ratio of photosynthetic CO_2_ assimilation and stomatal conductance (Farquhar et al., 1982, 1989; Condon et al., 2004; Pita et al., 2005). Variation in iWUE among and within tree species can be estimated by the analysis of carbon isotope composition (δ^13^C) in plant tissues (Condon et al., 2002; Pita et al., 2005). A strong positive correlation between iWUE and δ^13^C has been reported for numerous crop and tree species (e.g., Sun et al., 1996; Condon et al., 2004; Roussel et al., 2009). Measuring leaf δ^13^C has several conceptual and logistical advantages to screening for drought tolerance based on iWUE (i.e., *A*/*g*_*s*_). Leaf δ^13^C is an attractive parameter that provides a spatial and temporal measure of essential traits related to carbon gain and plant water use (e.g., photosynthesis and stomatal conductance). Plant δ^13^C has been often used as a screening tool in breeding programs to select genotypes with greater WUE and productivity under drought conditions (Richards and Condon, 1993; Dixon and Carter, 2019; Famula et al., 2019). A rapid, accurate and non-destructive method (i.e., high-throughput phenotyping) to identify drought-tolerant woody plants for future breeding programs is also required. A promising approach is the use of chlorophyll fluorescence for the early detection of drought stress responses (Fahlgren et al., 2015; Rapacz et al., 2019). The measurement of leaf chlorophyll fluorescence is a commonly used screening tool to estimate photosynthetic activity (Zhuang et al., 2020). Plants able to maintain photosynthetic activity under water-limiting conditions are associated with the development of abiotic-stress tolerance (Su et al., 2015).

Tree growth and survival are essential traits for tree breeding. The selection of tree species and genotypes with high WUE traits may not always increase tree productivity. For instance, negative genetic correlations between growth and δ^13^C sometimes leads to negative trade-offs between water use and growth. In conifers, Xu et al. (2003) reported a low to moderate positive genetic correlation between δ^13^C and height and diameter in *Araucaria cunninghamii Mudie*. Conversely, strong negative genetic correlations (−0.83 to −0.96) were found between tree height and δ^13^C in *Pinus caribaea* Morelet (Xu et al., 2000). Likewise, two studies on *Pinus taeda* L. found negative genetic correlations between tree height and δ^13^C (Baltunis et al., 2008; Cumbie et al., 2011). Therefore, the assessment of the δ^13^C signature in addition to growth traits and yield improve the selection of drought-tolerant crops while maintaining productivity (Cregg and Zhang, 2001; Rebetzke et al., 2002; Condon et al., 2004; Gebrekirstos et al., 2011; Sinclair, 2011). To date, very few studies have reported the use of δ^13^C in tree improvement programs in *P. radiata*.

Genomic selection (GS) refers to the prediction of unobserved phenotypes using genome-wide genetic markers, such as single nucleotide polymorphisms (SNPs). Genomic information can be constructed from pedigree-based relationships among individuals to predict breeding values, known as Best Linear Unbiased Prediction (ABLUP), or from the construction of marker-based relationship matrices and mixed linear models, known as Genomic Best Linear Unbiased Prediction (GBLUP) (Wright, 1922; Meuwissen et al., 2001; VanRaden, 2008). As opposed to traditional quantitative genetics, the application of GS in breeding programs have far-reaching implications for the selection of drought-tolerance traits to reduce the time of progeny trials and improve the accuracy of selection (Grattapaglia and Resende, 2011; Thavamanikumar et al., 2013). A previous study has provided evidence that GS could shorten *P. radiata* breeding cycle from 17 to 9 years in New Zealand (Li and Dungey, 2018). In the present study, we investigated the use of genomic selection to study the genetic variation in drought-tolerance traits of 622 commercially-used *P. radiata* genotypes from 63 *P. radiata* families. Specifically, we applied the pedigree-based ABLUP and the marker-based GBLUP approaches to study the following objectives: (1) to determine the genetic variation (variance components and heritability estimates) of growth traits, δ^13^C and Y after exposure to water stress, (2) to estimate the genetic correlations of growth traits, δ^13^C and Y after exposure to water stress, and (3) to compare the accuracy of ABLUP and GBLUP breeding values and evaluate the relative selection efficiency of GBLUP over ABLUP in the future genomic selection of *P. radiata* in New Zealand.

## Materials and Methods

### *Pinus radiata* Genotypes and Measurement of Plant Traits

Plant material was sourced from a cloned elite population from the New Zealand Radiata Pine Breeding Company (RPBC). We selected a representative population of the third generation of the New Zealand *P. radiata* breeding program originated from the *Elite* germplasm collection (Dungey et al., 2009). The study included 622 genotypes from 63 families (10 genotypes per family) resulting from crossing among 55 parents, with an average of 30 clones per family. The *P. radiata* genotypes selected for this study displayed a wide range of variation in stem growth rate, wood density and pathogen resistance. Three ramets per genotype of similar height and basal diameter were used, with a total of 1,866 plants included in the study. Genotypes were vegetatively propagated from stool beds as bare-rooted cuttings. In August 2015, trees were transferred to 1-L pots with the industry-standard potting mix at 70% water holding capacity (WHC) and acclimated to the polyhouse conditions for over 9 months. The polyhouse was located at Scion’s nursery in Rotorua, New Zealand (Latitude −38°09′28.8′′S, Longitude −176°16′03.3E), and provided natural light but no temperature or humidity control. Plants in the polyhouse were not exposed to precipitation and thus plants were irrigated. The climate in the trial location is temperate, with the warmest months being in summer (January to March), and the coolest months being in winter (June to August). The mean daily maximum temperature is 27°C (summer), while the mean daily minimum temperature is 5°C (winter) (Chappell, 2014).

Pots were positioned within the polyhouse in an incomplete block design. Irrigation was provided by manual watering of pots twice a week, with the irrigation adjusted to maintain potting medium at 70% WHC before the water stress was applied. The experiment was conducted on three biological replicates of each genotype of similar height and basal diameter. Before the commencement of water stress (May 2016), the basal diameter (BD_I_) and height (H_I_) of each plant were measured. In addition, four fully developed expanded fascicles were collected from under the flushed buds of each ramet. Fresh needles were oven-dried at 65°C and cryo-ground before analyzing carbon isotopic composition (δ^13^C_I_) using continuous-flow isotope ratio mass spectrometry (Europa Scientific Ltd., Crewe, United Kingdom) in the Stable Isotope Unit at the University of Waikato (Hamilton, New Zealand). The δ^13^C values (‰) were expressed relative to the Vienna Peedee Belemnite standard (Craig, 1957):


(1) δ⁢C13⁢(‰)=Rs⁢a-Rs⁢dRs⁢d×1000

where *R*_*sa*_ and *R*_*st*_ are the ^13^C/^12^C ratios of the sample and the standard, respectively.

In early May 2016, three ramets per genotype of similar height and basal diameter were exposed to drought stress at 22–25% WHC. The amount of water required to reach 22–25% WHC was estimated based on the pot weight (including plant and medium). Over the course of the experiment, changes in pot weight due to tree growth were corrected using a linear relationship between pot weight and tree height. The water stress treatment was maintained by calculating the average weight change of 20–30 pots and compensating water loss every week for 11 months (late March 2017). After 6 months (November 2016) and 10 months (March 2017) of water stress, basal diameter (BD_6_ and BD_10_) and height (H_6_ and H_10_) of each plant were measured. An additional δ^13^C analysis in leaves was performed 10 months after water stress (March 2017, δ^13^C_10_). The maximum quantum yield of Photosystem II (PSII) in the dark-adapted state (Y) was measured after 5 months (October 2016, Y_5_) and 10 months (March 2017, Y_10_) of water stress using a pulse-amplitude modulated Portable Chlorophyll Fluorometer (Mini-PAM Photosynthesis Yield Analyzer, Heinz-Walz GmbH, Effeltrich, Germany). For each measurement, three ramets per genotype were assessed over 3 days to reduce the effect of the diurnal cycle. On the first day, maximum (*F*_*m*_) and minimum (*F*_*o*_) chlorophyll fluorescence emissions of dark-adapted needles were measured randomly in all ramets. During the two consecutive days, *F*_*m*_ and *F*_*o*_ measurements were repeated in a different order. The *Y* values for each plant were calculated as (*F_*m*_* − *F*_*o*_)/*F*_*m*_ and averaged across 3-day measurements. For each ramet, three Dark Leaf Clips (DLC-8) were simultaneously placed on mature, fully elongated needles. Needles were arranged flat across the opening of the leaf clip and the sliding shutter close 20 to 60 min before the measurement to ensure stable readings. Preliminary testing on a plant subset showed that diurnal variation was not significantly influencing the repeated Y measurements within the 3 days.

All replicate plants were carefully dissected after the 11 months of water stress into shoot and root samples. The potting medium was removed gently from the roots, and the root systems were then rinsed in running tap water. Biomass fractions, including shoot dry weight (SDW) and root dry weight (RDW) as well as total biomass or total dry weight (TDW), were determined after oven-drying the samples at 65°C for at least 72 h.

### Genomic Data

The genomic information for the 622 genotypes was obtained using the exome-capture genotyping by sequencing (GBS) method (Neves et al., 2013). Further details of SNP discovery and capture probe design and testing were previously described in Telfer et al. (2018, 2019). The total number of single nucleotide polymorphisms (SNPs) markers genotyped was 1,371,123. Markers with minor allele frequency (MAF) < 0.01 and missing marker data across genotypes > 40% were discarded from the analysis. The final number of SNP markers used in this study was 61,418. The individual missing SNP genotypes were substituted with the mean genotype for that SNP as implemented in *rrBLUP*-R package (Endelman, 2011).

### Statistical Models Used in the Genetic Analysis

Genetic analysis was computed with the Average Information Restricted Maximum Likelihood (REML) algorithm in the *ASReml-R v.3* R package (Butler et al., 2009). Single-trait analysis was performed to estimate variance components and heritability for each trait separately, whereas bivariate analysis was performed to estimate genetic correlations among traits. All genetic parameters were estimated and compared using a pedigree-based model best linear unbiased prediction (ABLUP) and genomic-based unbiased prediction (GBLUP).

Preliminary analyses were used for all traits to compare the performance of a spatial first-order autoregressive mixed model and a mixed model without the autoregressive term. The preliminary analyses showed that the spatial mixed model performed better than the mixed model without the autoregressive term (non-spatial) based on Akaike’s information criterion (AIC). Therefore, only spatial mixed models were used in the current study.

The single-trait pedigree-based analysis for all traits was performed using the following model:


(2) y=X⁢β+Z⁢a+Z⁢p⁢e+Z⁢b+e

where *y* is the vector of individual tree observations; β is the vector of fixed effects containing the overall mean; *a* is the random additive genetic effect which was assumed to be normally distributed ∼*N*$\left(0,A\,\sigma_{a}^{2}\right)$, where $\sigma_{a}^{2}$ is the additive genetic variance and *A* is the average numerator relationship matrix; *pe* is the vector of the random non-additive genetic effect of the individual ramet within the genetic material ∼*N*$\left(0,I\,\sigma_{p\,e}^{2}\right)$, where $\sigma_{p\,e}^{2}$ is non-additive genetic variance and *I* is the identity matrix; *b* is the vector of random incomplete block effect ∼N$\left(0,I\,\sigma_{b}^{2}\right)$, where $\sigma_{b}^{2}$ are the incomplete block variance and *I* is identity matrix; *X* and *Z* are incidence matrices of the fixed and random effects of the vector of phenotypes *y*; *e* is the random residual deviations of individual trees which was partitioned into a spatial (ξ) and independent (η) residuals as $\sigma_{\xi }^{2}$ [AR1 (*p*_*col*_) ⊗ AR1(*p*_*row*_)] + *I* σ^2^ where $\sigma_{\xi }^{2}$ is the spatial residual variance, ⊗ is the Kronecker product, and AR1(*p*) represents a first-order autoregressive correlation matrix for rows and columns where ρ is the autocorrelation parameter. The independent residual (η) was assumed to be pairwise independent as *I*σ^2^, where *I* is the identity matrix, σ^2^ is uncorrelated residual variance.

A bivariate analysis was employed to estimate the pairwise genetic correlations between each pair of traits. The variance-covariance structure for this model is:


$$\left[\frac{icb_{1}}{icb_{2}}\right]∼N\;\left[\begin{array}{cc} I\sigma_{b_{1}}^{2} & 0\\ 0 & I\sigma_{b_{2}}^{2} \end{array}\right],\left[\frac{a_{1}}{a_{2}}\right]∼N\;\left[\begin{array}{cc} A\sigma_{a_{1}}^{2} & A\sigma_{a_{1}a_{2}}\\ A\sigma_{a_{2}a_{1}} & A\sigma_{a_{2}}^{2} \end{array}\right],$$

(3) $$\left[\frac{pe_{1}}{pe_{2}}\right]∼N\;\left[\begin{array}{cc} I\sigma_{pe_{1}}^{2} & 0\\ 0 & I\sigma_{pe_{2}}^{2} \end{array}\right],\left[\frac{e_{1}}{e_{2}}\right]∼N\;\left[\begin{array}{cc} I\sigma_{e_{1}}^{2} & I\sigma_{e_{1}e_{2}}\\ I\sigma_{e_{2}e_{1}} & I\sigma_{e_{2}}^{2} \end{array}\right]$$

where *icb*_1_ and *icb*_2_ represent the incomplete block effects for 1st and 2nd trait; $\sigma_{b_{1}}^{2}$ and $\sigma_{b_{2}}^{2}$ incomplete block variances for the 1st and 2nd trait, a_1_ and a_2_ represent the breeding values for the 1st and 2nd trait; $\sigma_{a_{1}}^{2}$ and $\sigma_{a_{2}}^{2}$ are the additive genetic variances for the 1st and 2nd trait; σ_*a*_1_*a*_2__ is the additive genetic covariance among traits, *pe*_1_ and *pe*_2_ represent the non-additive genetic effects for the 1st and 2nd trait; $\sigma_{p\,e_{1}}^{2}$ and $\sigma_{p\,e_{2}}^{2}$ are the non-additive genetic variances for the 1st and 2nd trait, *e*_1_ and *e*_2_ represent the spatially independent residuals; $\sigma_{e_{1}}^{2}$ and $\sigma_{e_{2}}^{2}$ are the independent residual variances for the 1st and 2nd trait and; σ_*e*_1_*e*_2__ is the residual covariance among traits, and *I* is the identity matrix. The genetic correlations between traits (*r*_*a*_) were estimated as


(4) $$r_{a}=\frac{\sigma_{a_{1}\,a_{2}}}{\sqrt{\sigma_{a_{1}}^{2}\,\sigma_{a_{2}}^{2}}}$$

The GBLUP model was the same as the ABLUP model, substituting the average numerator relationship matrix *A* by the marker-based relationship matrix *G* implemented based on (Forni et al., 2011) as follows:


(5) G=(M-P)(M-P)′trace[(M-P)(M-P)]′/n

Where *M* is the marker matrix with genotypes coded 0, 1, and 2 for the alternative allele homozygote, heterozygote and reference allele homozygote, respectively, and *P* is a vector of twice the allele frequency, *trace*[(*M*−*P*)(*M*−*P*)′]is a trace of the matrix defined in the nominator and *n* is the number of markers (Forni et al., 2011).

The individual narrow-sense heritability (*h*^2^) is the proportion of phenotypic variance that is explained by the additive genetic variance (Falconer and Mackay, 1996) and was estimated as:


(6) $$h^{2}=\frac{\sigma_{a}^{2}}{\sigma_{a}^{2}+\sigma_{p\,e}^{2}+\sigma_{e}^{2}}$$

where $\sigma_{a}^{2}$ is the additive genetic variance, $\sigma_{p\,e}^{2}$ is non-additive genetic variance, and $\sigma_{e}^{2}$ is the spatially independent residual variance. The two heritability estimates are reported in this study regarding implemented methods: $h_{a}^{2}$, a narrow-sense heritability using variance components estimated in ABLUP and $h_{g}^{2}$, a narrow-sense heritability using variance components estimated in GBLUP.

### Cross-Validation and Accuracies of Estimated Breeding Values

The cross-validation was conducted by examining the accuracy of breeding values obtained by the ABLUP (ABLUP-EBV) and GBLUP (GBLUP-EBV) models. The accuracy of both models was evaluated using 10-fold cross-validation with ten random replicates. All data were randomly divided into ten equal groups. In each replication, phenotypes from one group were masked and used as a validation dataset. The accuracies of breeding values obtained by estimating the average Pearson’s correlation between predicted breeding values from masked phenotypes and the breeding values estimated using the full dataset obtained from the ten replicates. These breeding values were obtained from the single-trait analysis (Eq. 2).

### Selection Response With Genomic Selection

Response to selection was calculated as the ratio between selection accuracy and breeding cycle length in years (Chen et al., 2018). Thus relative efficiency (RE) of GBLUP over ABLUP is:


(7) $$RE\left(\%\right)=\frac{r\,\left(G\,B\,L\,U\,P-E\,B\,V_{V},A\,B\,L\,U\,P-E\,B\,V_{F}\right)}{r\,\left(A\,B\,L\,U\,P-E\,B\,V_{V},A\,B\,L\,U\,P-E\,B\,V_{F}\right)}\times 100$$

Therefore, the relative efficiency of GBLUP over ABLUP per year is:


(8) $$R\,E\,y\,e\,a\,r^{-1}=\frac{r\,\left(G\,B\,L\,U\,P-E\,B\,V_{V},A\,B\,L\,U\,P-E\,B\,V_{F}\right)}{r\,\left(A\,B\,L\,U\,P-E\,B\,V_{V},A\,B\,L\,U\,P-E\,B\,V_{F}\right)}\times \frac{L_{T\,S}}{L_{G\,S}}\times 100$$

Where *GBLUP-EBV_*V*_* is the genomic breeding values for the trees in the validation dataset, *ABLUP-EBV_*F*_* is the estimated breeding values from the full dataset, *ABLUP-EBV_*V*_* is the estimated breeding values for the trees in the validation dataset, *L*_*TS*_ is the length of the breeding cycle for selection based on ABLUP which is approximately 17 years, and *L*_*GS*_ is the length of the breeding cycle for selection based on GBLUP, which is approximately 9 years (Li and Dungey, 2018).

The effective population size *Ne* (Wright, 1931, 1933) in the current study was 23 and was estimated based on the status number concept (Ns) of Lindgren et al. (1997) as:


(9) N⁢s=0.5/f

where Ns is the status effective number and *f* is the average coancestry of the population including the coancestry of individuals with themselves.

### Genotypic Variation in δ^13^C, Y, and Growth

One-way analyses of variance (ANOVAs) were conducted, after confirming data met assumptions for normality and homogeneous variance, to test whether means for drought-tolerant traits (i.e., δ^13^C and Y) differed between tree families and genotypes. A correlation was also assessed to test for the relationship between δ^13^C and Y after 10 months of water stress.

## Results

### Descriptive Statistics

Trees showed an average 39% increase in the growth of basal diameter and a 63% increase in height growth after 10 months of water stress. The mean δ^13^C value before water stress was −29.67‰, with a range of −26.16‰, to −32.29‰, and a standard deviation of 1.29‰ (Table 1). After 10 months of water stress, the mean δ^13^C value 26.50‰ with a range from −23.27‰ to −31.53‰ (Table 1). The mean *Y*-value for month five (*Y*_*5*_) was 0.79 with a range of 0.58–0.87 (Table 1). The mean *Y*-value at month 10 (*Y*_*10*_) was 0.77 with a range from 0.30 to 0.84 (Table 1).

**TABLE 1:** Number of observations (*N*), mean standard deviation (SD), minimum (Min), maximum (Max), and coefficient of variation (CV%) for the measured growth and drought-tolerance traits before, during and after water stress in *Pinus radiata* genotypes.

Trait	*N*	Mean	*SD*	Min	Max	CV%
^1^BD_I_ (mm)	1857	5.76	0.58	3.31	7.97	10
^2^BD_6_ (mm)	1821	7.55	0.88	4.5	10.5	12
^3^BD_10_ (mm)	1818	8.03	1.01	5.2	16.5	13
^4^H_I_ (cm)	1857	39.01	6.76	9	60	17
^5^H_6_ (cm)	1821	56.88	9.71	18	89	17
^6^H_10_ (cm)	1818	63.39	11.52	19	140	18
^7^δ^13^C_I_ (‰)	1846	–29.66	1.04	−32.29	−26.16	−3
^8^δ^13^C_10_ (‰)	1845	–26.49	1.69	−31.53	−23.27	−6
^9^Y_5_	607	0.79	0.029	0.581	0.867	37
^10^Y_10_	1819	0.77	0.030	0.300	0.840	44
^11^RDW (kg)	1853	0.63	0.18	0.023	1.96	29
^12^SDW (kg)	1859	1.83	0.66	0.25	9.07	36
^13^TDW (kg)	1852	2.46	0.78	0.28	11.03	32

*^1^BD_I_, initial basal diameter before water stress; ^2^BD_6_, basal diameter after 6 months of water stress; ^3^BD_10_, basal diameter after 10 months of water stress; ^4^H_I_, initial height before water stress; ^5^H_6_, height after 6 months from water stress; ^6^H_10_, height after 10 months of water stress; ^7^δ^13^C_I_, initial needle ^13^C before water stress; ^8^δ^13^C_10_, final needle ^13^C after 10 months of water stress; ^9^Y_5_, maximum quantum yield of PSII after 5 months of water stress; ^10^Y_10_, maximum quantum yield of PSII after 10 months of water stress; ^11^RDW, root dry weight after harvesting; ^12^SDW, shoot dry weight after harvesting; ^13^TDW, total dry weight after harvesting.*

### Genetic Parameters

The measured traits showed a wide range of heritability estimates based on either ABLUP ($h_{a}^{2}$) and GBLUP ($h_{g}^{2})$ approaches, ranging from 0.07 to 0.44 (Table 2). Using the ABLUP, $h_{a}^{2}$ for BD decreased from 0.17 (BD_I_) to 0.12 (BD_10_). In contrast, water stress increased $h_{a}^{2}$ for height from 0.38 (H_I_) to 0.44 (H_10_). The $h_{a}^{2}$ for δ^13^C_I_ was 0.17 compared with 0.07 for δ^13^C_10_. The $h_{a}^{2}$ of chlorophyll fluorescence after 5 months of water stress (Y_5_) was close to zero due to the small number of observations recorded for this trait (*N* = 607). However, *Y*_10_ was 0.06. The $h_{a}^{2}$ for dry weight measures (i.e., SDW, RDW, and TDW) were similar of approximately 0.13.

**TABLE 2:** Estimates of variance components and heritability for growth traits, carbon isotope composition and chlorophyll fluorescence.

Traits	ABLUP				GBLUP			
	$\sigma_{p\,e}^{2}$	$\sigma_{a}^{2}$	$\sigma_{\text{e}}^{2}$	$h_{a}^{2}$	$\sigma_{p\,e}^{2}$	$\sigma_{g}^{2}$	$\sigma_{\text{e}}^{2}$	$h_{g}^{2}$
^1^BD_I_	0.12	0.06	0.16	0.17 (0.06)	0.10	0.05	0.16	0.16 (0.06)
^2^BD_6_	0.10	0.04	0.50	0.06 (0.03)	0.08	0.04	0.48	0.07 (0.03)
^3^BD_10_	0.06	0.11	0.74	0.12 (0.04)	0.045	0.09	0.70	0.11 (0.04)
^4^H_I_	11.50	15.41	14.04	0.38 (0.10)	5.73	14.2	15.08	0.41 (0.08)
^5^H_6_	5.90	32.60	40.46	0.41(0.10)	4.91	20.67	38.48	0.32 (0.06)
^6^H_10_	0	55.39	69.29	0.44 (0.03)	6.17	30.01	66.37	0.29 (0.06)
^7^δ^13^C_I_	0.08	0.11	0.47	0.17 (0.06)	0.03	0.12	0.45	0.20 (0.05)
^8^δ^13^C_10_	0.06	0.19	2.33	0.07 (0.03)	0.03	0.18	2.27	0.07 (0.03)
^9^Y_5_	0.0008	0.000004	0.00007	0.004 (0.05)	0.001	0	0.0001	0
^10^Y_10_	0.00004	0.00006	0.0009	0.06 (0.03)	0.00004	0.00004	0.00096	0.04 (0.03)
^11^RDW	0.0003	0.0039	0.0251	0.13 (0.04)	0	0.0034	0.024	0.12 (0.02)
^12^SDW	0.006	0.05	0.35	0.12 (0.04)	0	0.04	0.34	0.11 (0.02)
^13^TDW	0	0.07	0.49	0.13 (0.03)	0	0.06	0.47	0.11 (0.02)

*Show are non-additive genetic variance ($\sigma_{p\,e}^{2}$), additive genetic variance$\left(\sigma_{a}^{2}\right)$ estimated from pedigree, and ($\sigma_{g}^{2})$ estimated by makers residual variance ($\sigma_{e}^{2})$, narrow-sense heritability ($h_{a}^{2}$) estimated using pedigree, and narrow-sense heritability ($h_{g}^{2}$) estimated using markers with approximate standard errors in parentheses for heritability estimates. ^1^BD_I_, initial basal diameter before water stress; ^2^BD_6_, basal diameter after 6 months of water stress; ^3^BD_10_, basal diameter after 10 months of water stress; ^4^H_I_, initial height before water stress; ^5^H_6_, height after 6 months from water stress; ^6^H_10_, height after 10 months of water stress; ^7^δ^13^C_I_, initial needle ^13^C before water stress; ^8^δ^13^C_10_, final needle ^13^C after 10 months of water stress; ^9^Y_5_, maximum quantum yield of PSII after 5 months of water stress; ^10^Y_10_, maximum quantum yield of PSII after 10 months of water stress; ^11^RDW, root dry weight after harvesting; ^12^SDW, shoot dry weight after harvesting; ^13^TDW, total dry weight after harvesting.*

Heritability estimates based on GBLUP were generally lower than the ABLUP estimates except for δ^13^C_I_ and H_I_, which showed slightly higher $h_{g}^{2}$values than $h_{a}^{2}$. The greatest difference in $h_{g}^{2}$ after water stress was recorded for tree height, in which the estimate decreased from 0.41 (H_I_) to 0.29 (H_10_).

### Genetic Correlations Between Traits

**FIGURE 1:**
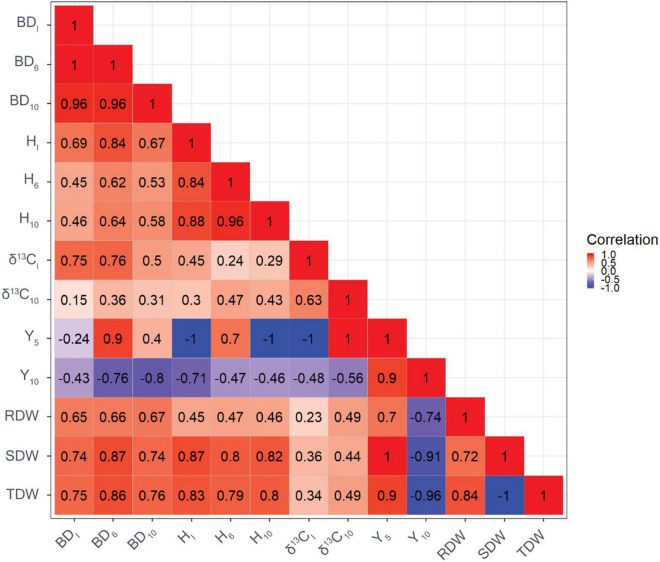
Genetic correlations among growth traits and carbon isotope composition δ^13^C based on ABLUP (the reddish the color, the greater value of genetic correlation). BD_I_ is the initial basal diameter before water stress, BD_6_ is basal diameter after 6 months of water stress, BD_10_ is basal diameter after 10 months of water stress, H_I_ is initial height before water stress, H_6_ is height after 6 months of water stress, H_10_ is height after 10 months of water stress, δ^13^C_I_ is initial needle δ^ 13^C before water stress, δ^13^C_10_ is final needle δ^13^C after 10 months of water stress, Y_5_ is maximum quantum yield of PSII after 5 months of water stress, Y_10_ is maximum quantum yield of PSII after 10 months of water stress, RDW is the root dry weight after harvesting, SDW is shoot dry weight after harvesting, and TDW is total dry weight after harvesting.

**FIGURE 2:**
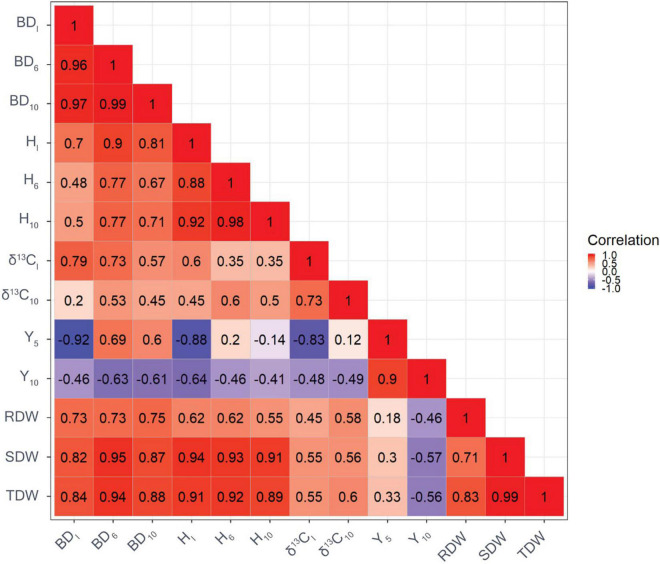
Genetic correlations among growth traits, carbon isotope composition δ^13^C based on GBLUP (the reddish the color, the greater value of genetic correlation). BD_I_ is the initial basal diameter before water stress, BD_6_ is basal diameter after 6 months of water stress, BD_10_ is basal diameter after 10 months of water stress, H_I_ is initial height before water stress, H_6_ is height after 6 months of water stress, H_10_ is height after 10 months of water stress, δ^13^C_I_ is initial needle δ^13^C before water stress, δ^13^C_10_ is final needle δ^13^C after 10 months of water stress, Y_5_ is maximum quantum yield of PSII after 5 months of water stress, Y_10_ is maximum quantum yield of PSII after 10 months of water stress, RDW is the root dry weight after harvesting, SDW is shoot dry weight after harvesting, and TDW is total dry weight after harvesting.

The genetic correlation between BD_I_ and BD_10_ was very strong (Figure 1). Similarly, a strong genetic correlation was found between H_I_ and H_10_ (*r* = 0.92). There were a high genetic correlations with H and BD at the beginning and end of the experiment. Genetic correlations between H and BD were moderate to large and ranged from 0.48 to 0.90. Moderate to high genetic correlations were found between biomass and tree growth traits, ranging from 0.54 to 0.99. The genetic correlation between δ^13^C_I_ and δ^13^C_10_ was 0.73. In addition, genetic correlations between δ^13^C and growth traits were generally positive, with the strongest correlation found between δ^13^C_I_ and BD_I_ (*r* = 0.79). Moderate genetic correlations were observed between δ^13^C and biomass traits, ranging from *r* = 0.45 to 0.60 regardless of the point in time. The genetic correlation between Y_5_ and Y_10_ was strong (*r* = 0.90). Furthermore, strong and negative genetic correlations were observed between Y_5_ and BD_I_ (*r* = −0.92), and between Y_5_ and H_I_ (*r* = −0.88). Genetic correlations between Y_10_ and growth traits, and δ^13^C were moderate, ranging from *r* = −0.41 to −0.64. Genetic correlations between traits based on GBLUP showed a similar pattern but were of weaker strength compared with genetic correlations based on ABLUP (Figure 2).

### Cross-Validation and Accuracies of Estimated Breeding Values

The cross-validation estimated moderate to large accuracies of breeding values for all traits using all models (Table 3). The accuracy ABLUP-EBV for diameter ranged from 0.77 for BD_I_ to 0.83 for BD_6_, and height ranged from 0.67 for H_10_ to 0.70 for both H_I_, and H_6_. The accuracy for biomass traits reached 0.80. The accuracies for carbon isotope δ^13^C were 0.80 for δ^13^C_10_ and 0.78 for δ^13^C_I_, and for Y_10_ was 0.79. The accuracy of GBLUP-EBV was 7–26% lower than ABLUP-EBV. The accuracy of GBLUP-EBV for diameter ranged from 0.61 for BD_I_ to 0.65 for BD_10_, and height ranged from 0.59 for H_10_ to 0.65 for H_6_. The accuracy for biomass traits ranged from 0.66 for RDW to 0.69 for TDW. The accuracies for carbon isotope δ^13^C traits were 0.66 for δ^13^C_I_ and δ^13^C_10_, and for Y_10_ was 0.52.

**TABLE 3:** Accuracy of breeding values from single trait pedigree-based model, single trait genomic based model (10-fold cross-validation), relative efficiency of genomic selection (GBLUP) over quantitative genetic (ABULP) selection, and the relative efficiency per year.

Trait	^14^ABLUP	^15^GBLUP	^16^RE (%)	^17^RE year^−1^ (%)
^1^BD_I_	0.77	0.61	79	150
^2^BD_6_	0.83	0.62	75	141
^3^BD_10_	0.78	0.65	83	157
^4^H_I_	0.70	0.63	89	169
^5^H_6_	0.70	0.65	93	175
^6^H_10_	0.67	0.59	88	167
^7^δ^13^C_I_	0.78	0.66	84	159
^8^δ^13^C_10_	0.80	0.66	82	155
^9^*Y_5_	–	–	–	–
^10^Y_10_	0.79	0.52	66	124
^11^RDW	0.80	0.66	83	158
^12^SDW	0.80	0.68	85	161
^13^TDW	0.80	0.69	87	164

^1^BD_I_, initial basal diameter before water stress; ^2^BD_6_, basal diameter after 6 months of water stress; ^3^BD_10_, basal diameter after 10 months of water stress; ^4^H_I_, initial height before water stress; ^5^H_6_, height after 6 months from water stress; ^6^H_10_, height after 10 months of water stress; ^7^δ^13^C_I_, initial needle ^13^C before water stress; ^8^δ^13^C**_10_**, final needle ^13^C after 10 months of water stress; ^9^Y_5_, maximum quantum yield of PSII after 5 months of water stress; ^10^Y_10_, maximum quantum yield of PSII after 10 months of water stress; ^11^RDW, root dry weight after harvesting; ^12^SDW, shoot dry weight after harvesting; ^13^TDW, total dry weight after harvesting; ^14^ABLUP, quantitative genetics BLUP estimate of breeding value; ^15^GBLUP, genomic breeding values estimated by GBLUP; ^16^RE, relative efficiency of genomic selection over quantitative genetics BLUP selection; and ^17^RE year^−1^, relative efficiency per year. *No accuracy values were obtained for Y_5_ due to the small number of observations.

The RE % of the models showed increased efficiency of GBLUP over ABLUP models (Table 3). For diameter traits, GBLUP showed an increased efficiency that ranged from 75% for BD_6_ to 83% for BD_10_. For height traits, the efficiency ranged from 88% for H_10_ to 93% for H_6_. For carbon isotope δ^13^C traits, the efficiency was 84% for δ^13^C_I_ and 82% for δ^13^C_10_, and for biomass traits, the efficiency ranged from 83% for RDW to 87% for TDW. The lowest relative selection efficiency using GBLUP was obtained for Y_10_ (66%). On the other hand, the relative efficiency in selection per year (RE year^−1^) resulted in the overall superiority of GBLUP over ABLUP. When assuming the reduction of time for progeny trials from 17 to 9 years by the genomic selection, the lowest relative selection efficiency per year using GBLUP was obtained for Y_10_.

### Relationship Between δ^13^C and Y in Response to Water Stress

**FIGURE 3:**
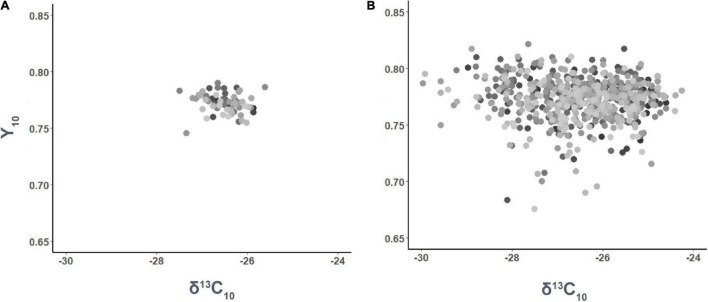
Phenotypic relationship between needle carbon isotope composition (δ^13^C_10_) and maximum quantum yield of PSII (Y_10_) after 10 months of water stress by family **(A)** and genotype **(B)**.

Significant differences in δ^13^C_I_ were only observed among families (*p* < 0.001), but not among genotypes (*p* = 0.193). In contrast, differences in δ^13^C_10_ were significantly influenced by both family (*p* = 0.002) and genotype (*p* = 0.005). On the other hand, Y_5_ differed among families and genotypes (*p* < 0.001 and *p* < 0.001, respectively), as well as Y_10_ (*p* < 0.001 and *p* < 0.001, respectively). Nonetheless, no significant linear relationship was found between δ^13^C_10_ and Y_10_ (*p* > 0.05) by family (Figure 3A) or genotype (Figure 3B).

## Discussion

### Genetic Parameters for Growth Traits and Foliar Carbon Isotope Composition

In this study, growth traits such as tree diameter and height presented moderate heritability after water stress. In case of tree diameter, heritability estimates decreased with time after water stress (0.17–0.12). This decrease was mainly due to the increasing residual variance from BD_I_ to BD_10_, even though the additive variance increased during the experiment. In contrast, heritability for height increased with time after water stress (0.38–0.44), likely due to increasing the additive genetic variance in balance with increasing residual variance. The observed heritability estimates for BD_I_ and H_I_ were in agreement with previous *in situ* field genetic studies in *P. radiata* across Australia and New Zealand sites (Kumar et al., 2008; Gapare et al., 2012), which reported heritability estimates between 0.10 and 0.48 for tree diameter, and 0.34 and 0.55 for height. Another study using two populations of water-stressed *P. radiata* seedlings under glasshouse conditions reported similar initial height estimates but lower initial diameter estimates (Espinoza et al., 2014).

We observed that average δ^13^C increased after water stress and the magnitude of the shift is similar to previous studies with water-stressed *P. radiata* (Walcroft et al., 1997; Korol et al., 1999; Barbour et al., 2002; Waghorn et al., 2015). The increase in δ^13^C under water stress had overall low to moderate effect on heritability estimates of growth traits before and after water stress, however, the heritability estimate for δ^13^C_I_ was twice than that for δ^13^C_10_. This is presumably due to the greater residual variance obtained for δ^13^C_10_ which was close to fivefold the residual variance for δ^13^C_I_. To our knowledge, no published studies investigated estimates for the δ^13^C in genotypes with narrow-sense heritability, such as *P. radiata* in New Zealand. In other conifers, low to moderate heritability estimates for δ^13^C have been reported; for instance 0.17 for *P. pinaster* (Brendel et al., 2002) and 0.09 for *P. taeda* (Baltunis et al., 2008). The decrease in δ^13^C heritability after the application of water stress has been observed in other studies across different species. Xu et al. (2003) found a reduction of between 0.33 and 0.20 in *A. cunninghamii* growing in wet sites compared to dry sites. Similarly, Ehdaie and Waines (1994) reported a lower δ^13^C heritability estimate in wheat-growing under drought conditions (0.12) compared to wet conditions (0.57). The authors justified this decrease due to the reduced additive genetic variance estimated under water stress conditions. Additionally, Klisz et al. (2019) found loss of any genetic signal in productivity among different Norway spruce provenances when these were growing at marginal climate conditions. Although it is difficult to make direct heritability comparisons across plant species, the results from these and other studies suggest that the induction of moderate to severe water stress may result in the loss of genetic variance in traits.

The estimates of additive genetic variance and narrow-sense heritability showed greater precision when GBLUP was implemented compared to ABLUP. Such a trend was also found in previous studies across different tree species (Gamal El-Dien et al., 2015, 2016; Chen et al., 2018). In summary, when using the marker-based genomic approach, heritability estimates decreased in most of the measured traits compared with the pedigree-based approach. These differences between the two types of correlations may be due to the fundamental differences between genomic and pedigree relationship matrices. For instance, the relationships in the pedigree-based relationship matrix are based on identity by descent (the probability that 2 alleles come from a common ancestor), whereas, the relationships in the genomic matrix reflect the identity by state probabilities (the probability that 2 alleles are the same) (Powell et al., 2010). Furthermore, differences between pedigree- and genomic-based correlations could also be driven by the source of information used. The former uses expected genetic covariation while the latter uses genetic covariation captured by SNPs (Veerkamp et al., 2011; Momen et al., 2017). Another explanation could be that unknown environmental effects were confounding the pedigree relationship-matrix and inflated the genetic variance (Lee et al., 2010).

### Genetic Correlations Between Plant Growth Traits and Water-Use Efficiency Measured as δ^13^C

This study recorded positive genetic correlations among δ^13^C, chlorophyll fluorescence, basal diameter and height growth, and biomass. The moderate to large genetic correlations between δ^13^C and growth traits, including tree height, diameter and biomass, suggest that fast-growing genotypes under water stress could display greater WUE. However, further studies of genetic correlations between δ^13^C and growth traits in *P. radiata* are warranted to confirm these results.

The findings of this study confirm previous studies with other species that found moderate to strong genetic correlations (Johnsen et al., 1999; Xu et al., 2000; Baltunis et al., 2008). The moderate genetic correlation obtained between the intital diameter or dimater measured after 6 months with initial δ^13^C suggests that diameter might be an indicator trait for greater WUE of *P. radiata* breeding populations. The positive genetic correlation between growth and δ^13^C_I_ or δ^13^C_10_ provides evidence that the variation in δ^13^C (a surrogate of WUE) between genotypes may be possibly due to the differences in the carboxylation efficiency of photosynthesis, rather than changes in stomatal conductance. Further, more drought-tolerant genotypes (i.e., more negative δ^13^C values) may be less sensitive to water stress and able to maintain photosynthetic capacity required for growth with longer periods of open stomata compared with less drought-tolerant genotypes. Rodríguez-Gamir et al. (2019) found that more water-efficient genotypes may be able to maintain a higher whole-plant hydraulic conductance under water stress, thus maintaining leaf water potential and able to keep the stomata opened for longer for photosynthesis. The same study also found that the down-regulation of root aquaporins is an important factor driving intraspecific changes in hydraulic conductance on *P. radiata* under water stress. Katul et al. (2003) and Rodriguez-Gamir et al. (2020) hypothesized that an equilibrium exists between maximum carbon demand by photosynthesis and maximum water supply, which might result in constant long-term mean intercellular CO_2_ concentration. This may be due to the co-evolution of hydraulic and biochemical plant attributes at the genotype level.

The genetic correlations between diameter or height before and after water stress were strong (0.88–0.99), indicating that genotypes were ranked consistently before and after imposing water stress. These strong genetic correlations indicate that the level of water stress in the current experiment was not strong enough to change the ranking of genotypes. This may be due to the experiment not applying severe enough water stress. Indeed, it is possible that genotype rankings could have changed using lower WHC, which would place the genotypes under more severe water stress deficit. Another theory is that only a few genotypes were sensitive to the water stress. However, results indicate that the best performing genotypes for growth traits will remain the best under water stress conditions, which is important for *P. radiata* breeding programs.

Negative genetic correlations were obtained in this study between δ^13^C and Y. That is, genotypes that performed better under water stress presented more negative foliar δ^13^C values and larger Y values. Physiologically, these results suggest that more drought-tolerant genotypes are more efficient at photosynthesising and have more intact chloroplasts and chlorophyll (i.e., Photosystem II) under water stress, as suggested by recent studies in *P. radiata* genotypes (Rodríguez-Gamir et al., 2019; Rodriguez-Gamir et al., 2020). Further, Gallart et al. (2019) suggested that the superior growth performance of some *P. radiata* genotypes under dry field conditions could be due to genotype-specific responses to water stress. However, future research is required to test whether greater drought tolerance also involves greater photosynthetic rates over a wide range of genotypes (Maxwell and Johnson, 2000), to determine the strength of the relationship between Y and δ^13^C.

We did not observe a significant linear relationship between δ^13^C and Y traits. Although previous studies reported δ^13^C to be negatively associated with Y (Mena-Petite et al., 2000, 2005; Dias and Brüggemann, 2010), Y was a poor predictor of water stress tolerance across the 63 families we tested. The poor predictability of Y in our study could be due to several reasons. First, plants were under moderate rather than severe water stress. Unlike other studies, this study maintained the potting medium to a constant 22–25% WHC. The strong relationship between δ^13^C and Y determined by Mena-Petite et al. (2005) was obtained from Y measurements collected over a wide range of foliar relative water potential (∼25–85% range) after withholding irrigation over a short-term period. However, a small number of *Y* values in this study were below 0.70. Interestingly, our study showed a range of relative water content (%) in needles between 70 and 90% (data not shown), which suggests that the application of more severe and/or more prolonged water stress treatments could have led to stronger Y differences among genotypes. Second, Y is a measure of maximum potential quantum yield of chloroplasts under Photosystem II, which may differ of the actual achievable quantum yield and photosynthesis (e.g., electron transfer rate) under ambient light and water stress conditions (e.g., Subrahmanyam et al., 2006; Baker, 2008; Wu et al., 2008). Although the use of light-adapted chlorophyll fluorescence may be more appropriate to measure quantum yield, the time required to conduct the measurements was considered unsuitable for this study.

### Cross-Validation and Accuracies of Estimated Breeding Values

The accuracies of GBLUP-EBV for growth traits under the conditions used in this experiment were consistent or greater than the prediction accuracies obtained for growth traits in other tree species, such as *Picea glauca* (Moench) Voss (Beaulieu et al., 2014; Gamal El-Dien et al., 2016), *P. pinaster* (Bartholomé et al., 2016; Isik et al., 2016); and *P. tadea* (Resende et al., 2012). Accuracies of δ^13^C_I_ and δ^13^C_10_ could not be compared with literature because this is the first study that evaluates the accuracy of genomic prediction for WUE assessed by δ^13^C for *P. radiata*. Although the accuracy of estimated GBLUP-EBV was lower than the ABLUP-EBV, the relative efficiency of selection per unit of time (RE year^–1^) was greater, reaching half of the breeding cycle (Isik, 2014; Li and Dungey, 2018). This is consistent with the previous studies for height and diameter in *P. taeda* and *Picea abies* Karst, in which the relative efficiency of genomic selection per unit of time ranged from 53 to 181% (Resende et al., 2012; Chen et al., 2018).

In the current study, we used the status number concept (Ns) to estimate the effective population size (Ne). Tambarussi et al., 2019 found that the status number is equivalent to the effective population size only when the size of the population is constant over generations.

## Conclusion

We’ve demonstrated large genetic variation of *P. radiata* drought tolerance assessed by plant growth and needle δ^13^C (a surrogate trait for WUE). The needle δ^13^C had low to moderate heritability estimates, however, there were moderate to high genetic correlations with plant growth or biomass traits. Thus, genotypes of *P. radiata* with a narrow-genetic background can have significant physiological differences related to water-use efficiency traits. Our results suggest that the variation of needle δ^13^C could be primarily controlled by the photosynthetic capacity and photosynthesis, being the main process driving tree growth under water stress conditions. The results of this study have important practical implications for managing commercial *Pinus* forests across the globe: First, selection of family and genotype with both greater WUE and better plant growth appears to be practically possible without trade-off. This means that breeding programs using these traits should ensure that *Pinus* forest productivity is maintained or even improved under some degree of drought stress. Second, the genomic selection for WUE using δ^13^C is possible with a good degree of accuracy. Carbon stable isotopes are a useful tool to increase the agility of global breeding programs to respond to the challenges of climate change.

## Data Availability Statement

The datasets presented in this study can be found in online repositories. The names of the repository/repositories and accession number(s) can be found below: https://doi.org/10.5281/zenodo.5768774 (Ismael et al., 2021).

## Author Contributions

AI analyzed the data and wrote the manuscript. HD, JX, DM, and YL conceived and supervised the study, made substantial contributions to the interpretation of the results, and contributed to the revision of the manuscript. K-TB, PB, and MG-G collected the data. MG and JK made substantial contributions to the interpretation of the results and contributed to the revision of the manuscript. All authors contributed to the article and approved the submitted version.

## Conflict of Interest

AI was employed by Livestock Improvement Corporation (LIC), Hamilton, New Zealand, and Scion, New Zealand. DM, JX, JK, YL, PB, MG, K-TB, ET, and HD were employed by Scion, New Zealand. The remaining author declare that this study received funding from NZ Ministry of Business, Innovation and Employment and the Radiata Pine Breeding Company. The funders were not involved in the study design, analysis, interpretation of data, the writing of this article or the decision to submit it for publication.

## Publisher’s Note

All claims expressed in this article are solely those of the authors and do not necessarily represent those of their affiliated organizations, or those of the publisher, the editors and the reviewers. Any product that may be evaluated in this article, or claim that may be made by its manufacturer, is not guaranteed or endorsed by the publisher.
